# Superior haplotypes towards the development of blast and bacterial blight-resistant rice

**DOI:** 10.3389/fpls.2024.1272326

**Published:** 2024-02-28

**Authors:** Shamshad Alam, Krishna Tesman Sundaram, Uma Maheshwar Singh, Madamshetty Srinivas Prasad, Gouri Sankar Laha, Pallavi Sinha, Vikas Kumar Singh

**Affiliations:** ^1^ Rice Breeding Innovation, International Rice Research Institute (IRRI) South Asia Hub, Hyderabad, India; ^2^ Rice Breeding Innovation, International Rice Research Institute-South Asia Regional Centre (ISARC), Varanasi, Uttar Pradesh, India; ^3^ Crop Protection, Indian Council of Agriculture Research-Indian Institute of Rice Research (ICAR-IIRR), Hyderabad, India

**Keywords:** rice, blast, BLB, GWAS, *haplo-pheno*, superior haplotype

## Abstract

Rice blast and bacterial leaf blight, are major disease, significantly threatens rice yield in all rice growing regions under favorable conditions and identification of resistance genes and their superior haplotypes is a potential strategy for effectively managing and controlling this devastating disease. In this study, we conducted a genome-wide association study (GWAS) using a diverse set of 147 rice accessions for blast and bacterial blight diseases in replications. Results revealed 23 (9 for blast and 14 for BLB) significant marker-trait associations (MTAs) that corresponded to 107 and 210 candidate genes for blast and BLB, respectively. The *haplo-pheno* analysis of the candidate genes led to the identification of eight superior haplotypes for blast, with an average SES score ranging from 0.00 to 1.33, and five superior haplotypes for BLB, with scores ranging from 1.52cm to 4.86cm superior haplotypes. Among these, superior haplotypes *LOC_OS12G39700-H4* and *LOC_Os06g30440-H33* were identified with the lowest average blast scores of 0.00-0.67, and superior haplotype *LOC_Os02g12660-H39* exhibited the lowest average lesion length (1.88 - 2.06cm) for BLB. A total of ten accessions for blast and eight accessions for BLB were identified carrying superior haplotypes were identified. These haplotypes belong to aus and indx subpopulations of five countries (Bangladesh, Brazil, India, Myanmar, and Pakistan). For BLB resistance, eight accessions from six countries (Bangladesh, China, India, Myanmar, Pakistan, and Sri Lanka) and four subpopulations (aus, ind1A, ind2, and ind3) were identified carrying superior haplotypes. Interestingly, four candidate genes, *LOC_Os06g21040, LOC_Os04g23960, LOC_Os12g39700*, and *LOC_Os01g24640* encoding transposon and retrotransposon proteins were among those with superior haplotypes known to play a crucial role in plant defense responses. These identified superior haplotypes have the potential to be combined into a single genetic background through haplotype-based breeding for a broader resistance spectrum against blast and bacterial blight diseases.

## 1 Introduction

Rice is a crucial global crop, with over 90% of its production and consumption concentrated in the Asian region. To meet the continuous demand for rice and feed the growing population, it is projected that we need to produce 25% more rice by the year 2030. However, the potential yield of the rice crop is significantly impacted by various pests and diseases. According to Savary et al. (2000), pests and diseases are responsible for causing yield losses of up to 41% annually in rice crops across Asia. The economic impact of these plant diseases is staggering, with over $220 billion lost due to diseases and at least $70 billion lost to invasive insects every year (FAO, 2019). Moreover, climate change is emerging as a major challenge for sustainable rice production. Shifting weather patterns brought about by climate change are significantly influencing the prevalence and severity of various plant diseases. Rising temperatures and increased carbon dioxide concentration are leading to physiological changes in plants, which in turn escalate the intensity of crop diseases. Disturbances in rainfall patterns and temperature are also affecting the life cycles, reproduction, and geographical distribution of many plant pathogens, further complicating disease management strategies. In light of these pressing challenges, the development of disease-resistant rice varieties that can withstand changing climate conditions becomes paramount.

Rice plants face severe attacks from various pathogens throughout their growth stages, significantly reducing yield and quality. Among these pathogens, Blast and Bacterial leaf blight (BLB), caused by *Magnaporthe oryzae* and *Xanthomonas oryzae*, respectively, are major diseases posing a substantial threat due to their wide distribution and potential damage under favorable conditions. Blast fungus infects rice at different growth stages, leading to an annual yield loss of approximately 10-30% (Devanna et al., 2022). It is particularly devastating in lowland rice in temperate and subtropical Asia and upland rice in tropical Asia, Latin America, and Africa. On the other hand, Bacterial blight causes systemic infection during the maximum tillering stage, resulting in 20-40% yield loss (Yang et al., 2020). Susceptible cultivars can suffer yield losses of up to 100%, depending on factors such as weather conditions, variety, nitrogen fertilizer application, and early-stage infection (Yang et al., 2020). The resistance against these diseases is governed by either vertical or horizontal resistance. Vertical resistance is conferred by major genes that provide race-specific resistance, while horizontal resistance is governed by multiple genes offering partial resistance with delayed and reduced disease lesion development. Horizontal resistance provides broad-spectrum and race-non-specific resistance. Extensive research on the genetics of resistance has led to the identification of more than 100 R-genes and 500 QTLs for blast, along with over 45 BLB R-genes (Lin et al., 2018; Zhang et al., 2017a: Neelam et al., 2020). Among these, 31 blast resistance and eleven BLB genes have been cloned and characterized at the molecular level (Lin et al., 2018). However, relying solely on race-specific resistance can lead to the evolution of pathogens, posing a challenge for breeders to regularly identify new genes/QTLs and develop durable and broad-spectrum resistant rice varieties.

For decades, blast and BLB have been devastating diseases that result in significant yield penalties in rice. Consequently, the urgent need to breed rice varieties with enhanced resistance has been recognized (Khush, 2005). Recently, several resistant genes against blast and BLB have been utilized in breeding programs to improve both elite rice varieties and parental lines of hybrids through Marker-Assisted Selection (MAS) (Dixit et al., 2020; Singh et al., 2022; Janaki Ramayya et al., 2021). However, deploying a single resistance gene often leads to rapid breakdowns. In the Philippines, for instance, deploying *Xa7* and other genes (*xa5* and *Xa21*) individually resulted in resistance lasting only about three years (Vera Cruz et al., 2000; Webb et al., 2010). To achieve broad-spectrum and durable resistance, the practice of pyramiding more than one resistance gene has been recommended (Khanna et al., 2015; Xiao et al., 2016). In various breeding programs, combinations of 2-4 R genes have been extensively utilized to achieve durable resistance against prevalent pathotypes of blast and BLB (Yugander et al., 2017). A few reported gene pyramids include *Pi54* + *Pi1* (Jamaloddin et al., 2020), P*iz5* + *Pi54* (Singh et al., 2013), *Pi9* + *Pita* (Khanna et al., 2015), *Pi9* + *Pi2* + *Piz* (Chukwu et al., 2019), *Xa21*+ *xa13* (Jamaloddin et al., 2020), *xa5*+ *xa13*+ *Xa21* (Pradhan et al., 2015), *Xa4*+ *xa5*+ *Xa21* (Suh et al., 2015), x*a13*+ X*a21* (Swathi et al., 2019), and *Xa4*+ *xa5*+ *xa13*+ *Xa21* (Dixit et al., 2020). These combinations have demonstrated effective and durable resistance against both blast and BLB, showcasing their potential for enhancing resilience to these devastating diseases.

The emergence of new races of pathogens in the field poses a challenge, as they can overcome resistant rice varieties within a few years after their existence (Chen et al., 2009). Therefore, it is essential to regularly identify new donors with novel or superior alleles/haplotypes and incorporate them into resistance breeding programs. Global germplasm collections, such as the 3000-rice genome project (3,000 rice genomes project, 2014) , hold a wealth of natural genetic diversity for various biotic and abiotic stresses. Exploring such diverse genetic resources enables the identification of new donors with genes/QTLs and their superior haplotypes. Genome-wide association studies (GWAS) using high-density single nucleotide polymorphisms (SNPs) and haplotype analysis have proven valuable in detecting genetic variants and novel alleles associated with traits of interest, directly benefiting genomic-assisted breeding to improve commercial cultivars (McCouch et al., 2016; Varshney et al., 2005; Varshney et al., 2009). In recent studies, association mapping has been employed to dissect the genetic architecture of blast (Kang et al., 2016; Frontini et al., 2021) and BLB resistance (Dilla-Ermita et al, 2017; Jiang et al., 2021). For instance, Lu et al. (2021) identified 11 QTLs for BLB resistance in a sub-set of 340 lines from the 3K RG panel. Raboin et al. (2016) identified three loci associated with blast resistance in *Japonica* and *Indica* panels. Additionally, Li et al. (2019) identified 56 QTLs for blast resistance in the rice diversity panel 1, and Volante et al. (2020) found 14 Marker Trait Associations (MTAs) associated with blast resistance, with 11 accessions exhibiting high resistance levels through field and growth chamber screenings.

Considering the above facts, the primary objectives of the present study are to identify significant MTAs and their underlying candidate genes conferring resistance to blast and BLB, to discover superior haplotypes (SH), and develop markers to track these SH. Furthermore, the study aims to identify novel genetic donors possessing high levels of resistance against blast and BLB, contributing to the ongoing efforts to develop resilient rice varieties against these two devastating diseases.

## 2 Materials and methods

### 2.1 Selection of a core sub-set of 3K RGP

A meticulous process was employed to select representative genotypes from 33 diverse countries, encompassing various subgroups within the 3K rice genome dataset to develop a core sub-set from 3K-RGP. This process involved curating a set of 147 accessions (28 aus, 28 ind1A, 26 ind1B, 35 ind2, 15 ind3, 55 indx, 2 trop, 1 aro and 1 admix) that collectively represents all the subpopulation of 3K rice genome panel. These accessions were chosen to facilitate the evaluation of their resistance against blast and bacterial blight diseases. To measure the genetic diversity, an analysis was conducted utilizing 0.5 million SNPs. This involved employing the Unweighted Pair Group Method with Arithmetic Mean (UPGMA) algorithm, which computed genetic distances based on molecular markers. The outcomes of the analysis were utilized to construct a UPGMA tree, helping in the careful selection of genotypes for subsequent screening. The genetic diversity assessment confirmed the variability within the genotypes, reinforcing the suitability of the chosen core sub-set for further analysis. A core sub-set of 147 accessions of 3K RG Panel from 33 different countries which include 28 aus, 28 ind1A, 26 ind1B, 35 ind2, 15 ind3, 55 indx, 2 trop, 1 aro and 1 admix were used in this study for phenotyping blast and bacterial blight resistance.

### 2.2 Phenotyping for blast and bacterial blight resistance

#### 2.2.1 Phenotyping for blast resistance

All the genotypes of sub-set 3K RG Panel along with resistant (IR64 and Tetep) and susceptible (HR12) check were screened for their reaction to blast under control conditions in three replications at CERF (Control Environment Research Facility) IRRI-South Asia Hub, ICRISAT Campus, Hyderabad during wet season 2021 (Alam et al., 2017). A highly virulent *M. oryzae* isolate called SPI-40 collected from IIRR, Hyderabad, Telangana was used for screening (Madhan Mohan., 2011). 8-10 Seeds of each line were sown in portray following random complete block design (RCBD). Resistant and susceptible checks were also shown in each portray. 21 days old seedlings were inoculated with blast spores using a hand sprayer. Inoculated seedlings were incubated in the dark chamber with relative humidity >90% for 24hr at 26°C. After 24 hr. of incubation, all the seedlings were grown at 28/26°C Day/night temperature and 16/8-hr light/dark photoperiod for disease development. Observation of each line was recorded on 0–5 SES scale (IRRI (International Rice Research Institute), 1996) 7 days after inoculation. Scores 0–2 were considered resistant (R), 3 as moderately resistant (MR), and 4-5 as susceptible (S). The average blast score was used for the genome-wide association mapping. An average of three replications were used for the analysis.

#### 2.2.2 Phenotyping of bacterial blight resistance

To evaluate accession of sub-set of 3k RG panel for bacterial blight resistance, a highly virulent isolate of *Xanthomonas oryzae* pv. oryzae (Xoo) DX-020, belongs to pathotype-4 (Yugander et al., 2017) was used. Eight plants of each accession of sub-set of 3K RG panel along with checks were screened for their reaction to BLB under controlled conditions at the maximum tillering stage during wet season 2021 in three replications. The rice plants were inoculated with freshly prepared inoculum following the clip inoculation method with sterilized scissors (Kauffman et al., 1973). Five plants and five fully expanded leaves per plant were inoculated (2-3cm from the tip). The disease assessment was done three weeks after inoculation by measuring the lesion length in centimetres from the cut tip of inoculated leave. The mean of two fully expanded leaf from three replicate plants for each accession were taken for determining the reaction type. A plant was classified as resistant if the average lesion length was shorter than 5 cm, moderately resistant if the lesion was >5-10 cm, moderately susceptible if the lesion was >10-15 cm, and susceptible if the lesion was >15 cm (Korinsak et al., 2021)

### 2.3 Genetic diversity and population structure analysis

Genetic diversity and population structure was done using genome-wide 0.5 million SNP data points of all the accessions. The tree was constructed using neighbor-joining method and the calculated dissimilarity index. Structure admixture analysis was analyzed through admixture software with k=3 as cross-validation error shown low. A Neighbor-joining tree was constructed using TASSEL Software version 5.0. The Phylogenetic tree is visualized through an unrooted tree using treeio R package. The Principal Component Analysis is done using SNPRelate R Package and Genetic Diversity and Analysis of Molecular Variance (AMOVA) is done using poppr R package.

### 2.4 GWAS, favourable allele, haplotype analysis and haplo−pheno analysis

Genome wide association study was performed using more than 0.5 million SNPs and the average SES score of three replications for blast and the average lesion length of five BLB clipped leaves. The analysis was conducted using five different multilocus GWAS models, including mrMLM (Wang et al., 2016), FASTmrMLM, FASTmrEMMA (Tamba and Zhang, 2018 ), ISIS EM-BLASSO, and pLARmEB (Zhang et al., 2017b), using an R-based methodology (https://CRAN.R-project.org/). The criterion for significance to find peak associations (MTAs) with the target trait is a LOD score of ≥5. The phenotypic allele effect (ai) was determined by the formula given by Zhang et al., 2013 and the favorable alleles of each trait.

To identify the candidate genes underlying the MTAs, all annotated genes contained within 50kbp LD from the peak MTA of the *Oryza sativa* reference sequence (Os-Nipponbare-Reference-IRGSP-1.0; RAP database: http://rapdb.dna.affrc.go.jp/download/irgsp1.html) were taken into consideration. Additionally, known blast R genes or literature-based QTLs were compared to the sites of the significant MTAs (Sharma et al., 2012; Ashkani et al., 2016) and recent literature (Kang et al., 2016; Ma et al., 2015, 2015; Zhao et al., 2011; Xu et al., 2014; Su et al., 2015; Mgonja et al., 2016; Zhu et al., 2016; Jiang et al., 2020; Yang et al., 2022).

The total candidate genes were taken into the *haplo-pheno* analysis for the identification of superior haplotypes. Haplotype analysis was carried using in-house scripts in R programming. Then the analysis included One-way ANOVA with haplotype as a fixed factor. Subsequently, Duncan’s multiple range test (DMRT) and ANOVA were used to test the statistical significance among the mean of haplotype groups using the Agricolae package in R (De Mendiburu, 2018). All reported P values underwent adjustment using either the Benjamini-Hochberg FDR or the Bonferroni method when multiple comparisons were performed, and a statistically significant threshold of adjusted p < 0.05 was considered.

For the whole investigation, we used the ‘3K filtered’ SNP set from the SNP seek database. The following filtering criteria were used to generate the filtered from the Base SNP set: (1) alternative allele frequency at least 0.01, (2) fraction of missed calls per SNP at most 0.292, and this SNP set was previously present in the SNP seek database, which was directly used in this work (http://snp-seek.irri.org/download.zul). Only nonsynonymous SNPs and indels in the exon region that cause an amino acid change were taken into account in the haplotype analysis for the candidate locus. The SNP-seek database (http://snp-seek.irri.org/_download.zul) was used to gather data on haplotypes and their diversity in order to identify the best haplotype by classifying haplotypes according to the genotype trait means for the related genes. The R-package (https://CRAN.R-project.org/) was used to create boxplots with a significance threshold of p <= 0.05.

## 3 Results

### 3.1 Insights into genetic diversity and population structure of the panel

The SNP-based diversity analysis provided valuable insights into the diversity exhibited by the panel (**Supplementary Figure 1**). The admixture analysis showed a notable distinction at k=2, distinctly separating the indica and aus groups. The optimal population structure emerged at k=3, revealing population divergence within aus, ind1A, and ind1B (**Supplementary Figure 1A**). The structure analysis revealed that indx has a blending of all subpopulations, signifying a historical interplay of domestication and selection between indx and other clusters within the population. Remarkably, while the admixture analysis highlighted the optimal k value as 3, it couldn’t distinguish the subpopulations of ind1B and ind3. However, the cladogram analysis provided confirmed relationships and divergences within these populations (**Supplementary Figure 1B**). The cladogram, illustrating the genetic relationships between samples, suggested the findings of identity-by-descent and neighbor-joining tree construction methods. The cladogram unequivocally reinforced the admixture results, underlining how indx’s presence is a consequence of controlled selection and domestication of various varieties. Notably, indx accessions demonstrated a heightened relatedness between ind3 and ind1B, elucidating the dispersion of their unique diversity and their positioning within the broader subgroups of ind2 and ind1B.

Employing principal component analysis (PCA) on our population, we extracted 15% of the total variation, effectively representing two distinct clusters. These clusters shed light on the contributions of aus and indica lines to the population’s genetic makeup. Despite the relatively modest variance captured by the PCA analysis, the second cluster, attributed to indica lines, elucidates the divergence in allele composition in a blended manner (**Supplementary Figures 1C, D**). To further delve into genetic diversity, the poppr r package was utilized, as displayed in **Supplementary Table 1**. Among the subpopulations, ind1A displayed the highest Nei’s genetic distance at 0.34, while indx exhibited the lowest at 0.23. As for the Shannon diversity index, indx emerged with the highest value of 3.9, in contrast to ind1A, which recorded the lowest at 2.6. An examination of molecular variance unveiled that 87.74% of the variance exists within samples, while the remaining 12.25% of the variance is accounted for between the distinct population groups.

### 3.2 Evaluation of disease resistance in the sub-set of 3K-RGP

Each of the accessions within the 3K-RGP sub-set underwent comprehensive screening for resistance against blast and BLB in multiple replications. Disease reaction showed minimal variation across replications, so the average score was used for the subsequent analysis (**Supplementary Figure 2**). In terms of blast severity, the spectrum of severity scores spanned from 0, indicating high resistance (e.g., LALSAITA::IRGC 43915-1), to 5, indicative of high susceptibility (e.g., RTS 16::IRGC 8235-1). Among all accessions, 12.92% showed resistance with a severity score (SES) of 0-2, another 12.92% demonstrated moderate resistance with SES scores above 2 to 3, while the majority, comprising 72.78% of genotypes, exhibited susceptibility with SES scores above 3 to 5. The reference susceptible check HR12 recorded a score of 5, while the resistant check Tetep scored 0. Evidently, the phenotypic variation in blast severity was considerable among the genotypes within the panel, revealing a pronounced divergence (**Supplementary Figures 2A–D**).

In the case of BLB screening, all accessions were inoculated during the maximum tillering stage. Lesion length averages across the panel ranged from 1.16cm (e.g., SUWEON 311: IRGC 61890-1) to 27.2cm (e.g., BPI 76 NON SENSITIVE (GREEN)::IRGC 9790-1). The susceptible check TN1 exhibited a lesion length of 21.5 cm, while the resistant check IRBB60 displayed a length of 0.3 cm. Among the accessions, 17.07% displayed resistance with mean lesion lengths varying from 1.13 to 3.00cm. Additionally, 42% were moderately resistant, characterized by mean lesion lengths spanning 3.08 to 6.00cm. Around 14.58% were moderately susceptible, with mean lesion lengths ranging from 6.02 to 8.92cm, and 25% of genotypes showed susceptibility, their mean lesion lengths varying from 9.02 to 27.2 cm (**Supplementary Figures 2B, E, F**).

### 3.3 Unveiling significant MTAs through multi-locus GWAS

**Figure 1:**
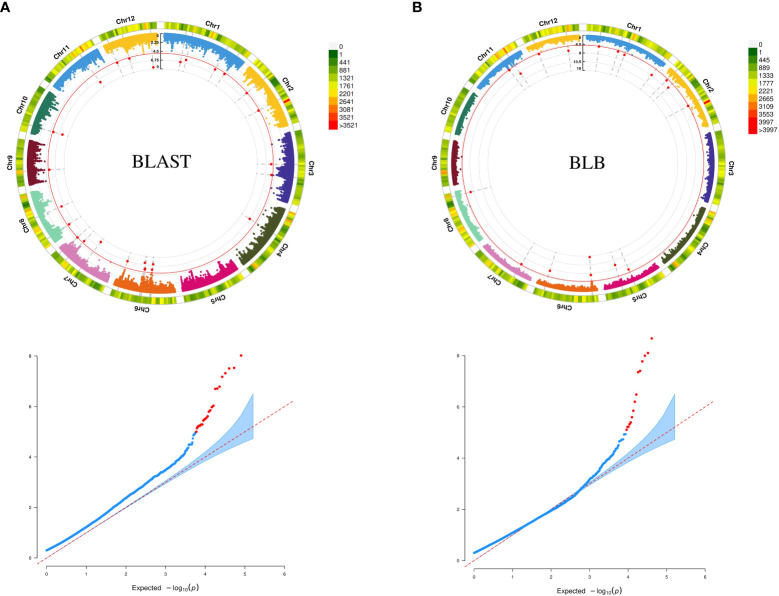
**(A)** Circular Manhattan and QQ-plot for MTAs associated with resistance to BLAST **(B) **Circular Manhattan and QQ-plot for MTAs associated with resistance to BLB.

We used GWAS analysis using 0.5 million SNPs from the selected accessions and their average scores against blast and BLB to unveil meaningful MTAs. Employing various multilocus GWAS methods (mrMLM, FASTmrMLM, and ISIS EM-BLASSO), we sought to identify significant associations by considering a LOD value ≥5 along with the phenotypic variance (PVE) as a threshold for significance. A total of 23 significant MTAs identified from the analysis, comprising nine for blast resistance and 14 for BLB resistance (**Table 1**). The soundness of the model is evident from the well-fitted Manhattan and Q-Q plots (**Figure 1**). The MTAs linked to blast resistance were dispersed across chromosomes 1, 4, 6, 7, 8, 11, and 12, with PVE ranging from 2.60% to 11.96%. Correspondingly, the BLB resistance revealed 14 MTAs, distributed across chromosomes 1, 2, 5, 6, 8, 11, and 12, encompassing PVE values ranging from 3.4% to 19.13%. These associations were identified through two distinct multi-locus GWAS models.

**Table 1:** MTAs significantly associated with resistance to Blast and BLB, PVE-Phenotypic variance explained, MTA) in red colour font are identified by more than one model.

Sl.no.	Trait name	Method	MTAs	Chromosome	Marker position (bp)	LOD score	‘-log10(P)’	r^2^ (%)	MAF	Allele	ai	Allele
1	Blast	mrMLM	10683327	1	10683327	5.9551	6.7866	4.8091	0.0862	C/T	-0.02	C
2	Blast	mrMLM	13878941	1	13878941	5.1707	5.9736	11.9621	0.2103	C/T	-0.55	T*
3	Blast	mrMLM	129276382	4	13654390	5.8709	6.6995	4.8875	0.0862	C/T	-0.45	T
4	Blast	mrMLM	198640034	6	17556914	6.4709	7.3193	4.8233	0.0724	G/A	0.01	G
5	Blast	FASTmrMLM	193194162	6	12111042	6.3317	7.1757	11.5096	0.1633	C/T	-0.88	T*
6	Blast	mrMLM	227131450	7	14799543	7.1513	8.0202	2.6068	0.0655	A/G	0.62	A
7	Blast	mrMLM	257538135	8	15508607	5.2239	6.0289	6.1712	0.2207	T/A	0.26	T*
8	Blast	mrMLM	337113150	11	20420593	6.6519	7.506	2.9458	0.0586	C/T	0.02	C
9	Blast	mrMLM	370184118	12	24470455	7.6345	8.5169	5.3386	0.0966	T/C	-0.24	C*
10	BLB	ISIS EM-BLASSO	7022436	1	7022436	5.7768	6.6021	3.4128	0.4948	T/A	-0.28	A
11	BLB	FASTmrMLM	21961294	1	21961294	5.3902	6.2015	4.0122	0.2539	C/A	-0.32	C
12	BLB	ISIS EM-BLASSO	41356309	1	41356309	7.7872	8.6736	6.9627	0.1021	C/A	0.09	C
13	BLB	FASTmrMLM	46203301	2	2932378	5.0514	5.8496	5.2263	0.2827	A/T	1.30	A
14	BLB	ISIS EM-BLASSO	49858526	2	6587603	6.914	7.776	4.1491	0.3429	T/G	-1.11	T
15	BLB	FASTmrMLM	165667744	5	14543058	5.6606	6.4818	7.9441	0.2225	G/C	-0.80	C*
16	BLB	FASTmrMLM & ISIS EM-BLASSO	172214131	5	21089445	10.0091	10.9476	8.3589	0.144	A/T	-1.20	A*
17	BLB	ISIS EM-BLASSO	183761269	6	2678149	16.3437	17.385	19.133	0.0916	T/A	-0.35	A*
18	BLB	FASTmrMLM	183835892	6	2752772	7.2317	8.1029	6.9823	0.089	T/G	0.30	T*
19	BLB	FASTmrMLM	207805809	6	26722689	7.1295	7.9978	4.7948	0.4319	T/C	-0.32	T
20	BLB	ISIS EM-BLASSO	268415807	8	26386279	6.5528	7.4038	3.6477	0.3168	C/T	-0.03	T
21	BLB	FASTmrMLM	335140024	11	18447467	11.1419	12.1027	13.384	0.1387	G/A	-0.56	A*
22	BLB	FASTmrMLM & ISIS EM-BLASSO	339202348	11	22509791	9.0862	10.0046	5.7834	0.1021	G/A	-0.36	G*
23	BLB	FASTmrMLM	366828503	12	21114840	6.5052	7.3547	6.5908	0.2932	G/A	0.43	A*

MAF, Minor Allele Frequency; QTN, Quantitative Trait Nucleotide; r^2^, Phenotypic variance explained, Marker Trait Association (MTA); ai, Phenotypic effects; ‘*’ denotes Favourable Allele.

The Phenotypic allele effect (ai) signifies the direction as the negative values denote to reduce the severity of the disease and positive effect increases in change. When selecting the MTA with positive allele effect it is better to select alternate allele as a faourable allele.

### 3.4 Prediction and annotation of potential candidate genes

A comprehensive search yielded 107 candidate genes from the nine significant MTAs associated with blast resistance. Similarly, for BLB, 210 candidate genes emerged from the 14 significant MTAs. To enhance our understanding, all identified candidate genes for both blast and BLB were annotated using the RAP-DB database (**Supplementary Table 2**)

### 3.5 GWAS findings aligned with known resistant genes

We compared the SNP positions of the prime MTAs pertaining to the target trait with those of established blast and BLB resistance genes or QTLs. By assessing candidate genes positioned within a range of −/+ 50kbp sequences from the peak SNP marker, we sought connections with known R-genes/QTLs. It’s noteworthy that all the significant MTAs for both blast and BLB were distinct, as there were no prior reports of R-genes or QTLs from these regions, except for the presence of the blast resistance gene *Pi40* (16274830–17531111) in close proximity to MTA-17556914 on chromosome 6. Nonetheless, several other genes and transcription factors associated with defense response were successfully identified. For instance, among the 107 candidate genes related to blast resistance, 18 corresponded with already documented genes associated with defense response mechanisms. In contrast, for BLB, a substantial number of 54 candidate genes aligned with previously reported defense response genes were identified. However, when we expanded the search to include sequences within −/+ 100 kb from the significant MTAs, we found four overlapping resistant genes on chromosome 11 associated with bacterial blight: *Xa23* (*LOC_Os11g37620*), *Xa39* (*LOC_Os11g37759*), *Xa10* (*LOC_Os11g37570*), and *Xa46* (*LOC_Os11g37540*). These findings contribute to a deeper understanding of the genetic basis of blast and bacterial blight resistance, offering valuable insights for the development of improved disease-resistant varieties through targeted breeding programs.

### 3.6 Identification of favourable SNP alleles linked to blast and BLB resistance

All MTAs associated with blast and BLB resistance underwent a detailed exploration to uncover favourable SNP alleles. This investigation categorized SNPs demonstrating a negative SNP effect (ai) leading to increased resistance against blast and BLB diseases as “favourable alleles.” Conversely, alleles resulting in positive SNP effect (ai) to blast and BLB diseases were classified as “unfavorable alleles. Allele with more than 5% PVE with high phenotypic allele effect (ai) were selected as favourable allele. For blast and BLB traits, MTAs showcasing a reduction in phenotypic allele effect were recognized as harboring favorable alleles. For instance, for blast resistance, (MTA-193194162 and MTA-13878941 exhibiting high PVE with 11.5% and 11.96%, carrying ‘T’ and ‘T’ alleles denoting a decrease in phenotypic allele effect by -0.88 and -0.56 respectively. Similarly, for BLB, (MTA-335140024 and MTA-183761269), possessing the ‘A’ and ‘A’ alleles respectively, demonstrated a reduction in phenotype by -0.56 and -0.35 with high PVE of 13.38% and 19.13%. However, in case of blast and BLB, a total of four and nine MTAs were identified respectively as favourable alleles with high PVE. This study effectively illustrated that genotype possessing favourable SNPs for the target trait exhibited a substantial decrease in the phenotypic effect of the trait (**Table 1**).

### 3.7 *Haplo−pheno* analysis for the identification of superior haplotypes

To determine the haplotype diversity, haplotype analysis was conducted on the 3K RG panel sub-set, correlating phenotypic outcomes against SNP data for all 317 identified candidate genes (107 for blast and 210 for BLB). This comprehensive analysis aimed to delineate the diversity of haplotypes within the 3K-RG panel. Significant haplotype diversity across 107 genes for blast and 210 genes for BLB were observed (**Supplementary Figure 5**).

**Figure 2:**
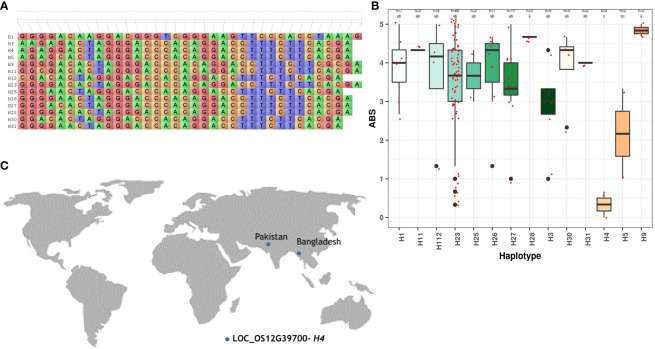
Haplotype analysis of *LOC_OS12G39700* across the sub-set panel. **(A)** Haplotypic variation of *LOC_OS12G39700*, a gene associated with blast resistance. **(B)** Boxplot showing variation in blast resistance among 147 accessions of 3K RGP **(C)** The geographical distribution of superior haplotype.

For a better comprehension of the phenotypic performance associated with specific haplotypes, the trait mean was calculated for each individual haplotype. The objective was to identify superior haplotypes, defined as those leading to significantly lower blast scores or lesion lengths for BLB compared to other haplotypes. Statistically validated by Duncan’s test, all candidate loci possessing more than two haplotypes were scrutinized to pinpoint superior haplotypes (**Table 2**). A number of superior haplotypes linked to resistance traits were successfully detected. In the case of blast, eight superior haplotypes were defined by blast scores ≤2. Among the identified superior haplotypes, eight candidate genes exhibited blast scores ranging from 0.00 to 1.33. *LOC_OS12G39700* and *LOC_Os06g30440* stood out with superior haplotypes H4 and H33, showing the lowest average blast scores of 0.00-0.67 (**Figure 2**; **Supplementary Figure 3**). Furthermore, the superior haplotype H29, associated with candidate gene *LOC_Os01g18910*, displayed an average blast score of 0.33-1.00. Similarly, H18 emerged as the superior haplotype for *LOC_Os01g24540, LOC_Os01g24640*, and *LOC_Os01g24750*, manifesting an average blast score within the range of 0.33-1.33. Additionally, candidate gene *LOC_Os04g23960* featured the superior haplotype H17, while *LOC_Os06g21040* harbored H177 and H60 as superior haplotypes with scores of 0.67-1.33.

**Figure 3:**
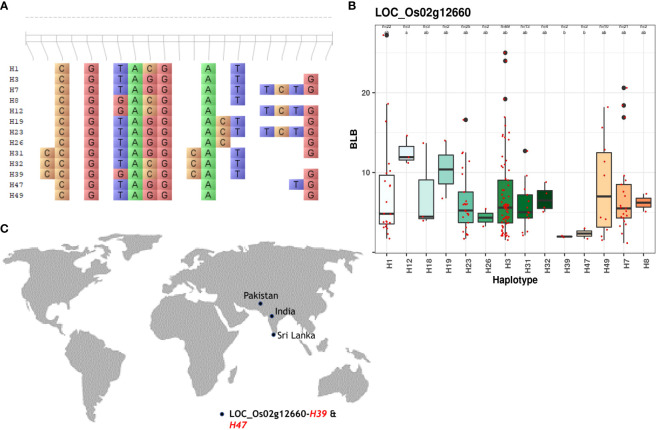
Haplotype analysis of *LOC_Os02g12660* across the sub-set panel. **(A)** Haplotypic variation of *LOC_Os02g12660*, a gene associated with BLB resistance. **(B)** Boxplot showing variation in BLB resistance among 147 accessions of 3K RGP **(C)** The geographical distribution of superior haplotype.

For BLB, superior haplotypes were characterized by average lesion lengths ≤3. Out of the 210 candidate genes analyzed, four genes located on chromosomes 2, 5, and 6 yielded superior haplotypes (**Table 2**). Among these genes, a total of five superior haplotypes with BLB scores spanning from 1.52cm to 4.86cm were identified. Notably, *LOC_Os02g12660* contained the superior haplotype H39, showcasing the lowest average lesion length of 1.88 – 2.06cm (**Figure 3**; **Supplementary Figure 4**). Identifying these superior haplotypes in the context of the studied traits holds promising implications for developing next-generation disease-resistant cultivars.

**Table 2:** Accessions having superior haplotypes for resistance to blast and BLB.

	Trait	Genotype	Genes	Superior haplotype	Average Blast Score^*^	Sub population	Origin
1	Blast	ARC_10100::IRGC_20709-1	*LOC_Os01g18910*	H29^c^	0.33	aus	India
2	Blast	PALEPYU::IRGC 33549-1	*LOC_Os06g21040*	H177^b^	1.00	indx	Myanmar
3	Blast	N 22::IRGC 46459-1	*LOC_Os06g21040*	H177^b^	1.00	aus	India
4	Blast	AUS 329::IRGC 29116-1	*LOC_Os01g24640*	H18^b^	1.33	aus	Bangladesh
			*LOC_Os01g24750*	H18^b^	1.33		
5	Blast	AUS 439::IRGC 29221-1	*LOC_Os01g24540*	H18^b^	1.00	aus	Bangladesh
			*LOC_Os01g24640*	H18^b^	1.00		
			*LOC_Os01g24750*	H18^b^	1.00		
6	Blast	CEA 3::IRGC 116965-1	*LOC_Os06g30440*	H33^c^	0.33	indx	Paraguae
7	Blast	INIAP 6::IRGC 117002-1	*LOC_Os04g23960*	H17^b^	0.67	aus	India
8	Blast	IRGA 959-1-2-2F-4-1-4A-6-CA-6X::IRGC 117006-1	*LOC_Os06g30440*	H33^c^	0.67	indx	Brazil
			*LOC_Os04g23960*	H17^b^	0.67		
9	Blast	LALSAITA::IRGC 43915-1	*LOC_OS12G39700*	H4^bc^	0.00	aus	Bangladesh
10	Blast	TAK::IRGC 73124-1	*LOC_Os06g30440*	H33^c^	0.67	aus	Pakistan
			*LOC_OS12G39700*	H4^bc^	0.67		
	Trait	Genotype	Genes	Superior haplotype	Average Lesion Length^#^	Sub population	Origin
1	BLB	HODARAWALA::IRGC 67631-1	*LOC_Os06g05940*	H112^b^	2.00	aus	Sri Lanka
3	BLB	MIN ZAO 6::IRGC 63772-1	*LOC_Os05g35490*	H49^c^	1.54	ind1A	China
4	BLB	KURULU WEE WHITE::IRGC 66518-1	*LOC_Os02g12660*	H39^b^	1.88	aus	Sri Lanka
5	BLB	SUFAID 246::IRGC 28303-1	*LOC_Os02g12660*	H39^b^	2.06	aus	Pakistan
6	BLB	YEBAWYIN::IRGC 33885-1	*LOC_Os05g35490*	H49^c^	2.34	ind3	Myanmar
7	BLB	NOROI::IRGC 31611-1	*LOC_Os06g05940*	H112^b^	3	aus	Bangladesh
8	BLB	KUTTA::IRGC 52184-1	*LOC_Os02g12660*	H47^b^	1.7	ind2	India

*Average blast score is recorded 0–5 SES scale (Standard Evaluation System, IRRI, 1996). Scores 0–2 were considered resistant (R), 3 as moderately resistant (MR), and 4-5 as susceptible (S). ^#^Average lesion length <5 cm is resistant, >5-10 cm is moderately resistant, >10-15 cm is moderately susceptible, and >15 cm susceptible (Korinsak et al., 2021). Different alphabet of small letters denoted in superscript is defined as significantly different groups which authors have got from duncan's test to perform haplo-pheno test.

### 3.8 Identification of accessions carrying superior haplotype

**Figure 4:**
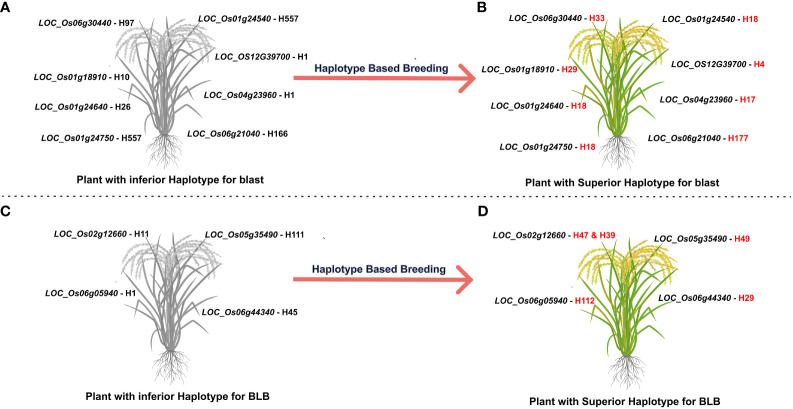
Towards developing tailored made rice with superior haplotypes for blast and BLB resistance. **(A, C)** The most inferior haplotype combination for blast and BLB resistance **(B, D)** the most superior haplotype combination for enhanced blast resistance. Through haplotype-based breeding, new breeding lines can be developed with the most superior haplotype combination.

A selection of 10 accessions was successfully identified carrying superior haplotypes corresponding to eight candidate genes associated with blast resistance. These accessions fall into the aus and indx subpopulations across five distinct countries: Bangladesh, Brazil, India, Myanmar, and Pakistan. For instance, accession AUS 439:IRGC 29221-1 (*LOC_Os01g24540*-H18, *LOC_Os01g24640-*H18, and *LOC_Os01g24750-*H18) was recognized as bearing the superior haplotype for three candidate genes:. Similarly, two accessions, namely AUS 329:IRGC 29116-1 (*LOC_Os01g24640-*H18, *LOC_Os01g24540-*H18) and TAK::IRGC 73124-1 (*LOC_Os06g30440*-H33, and LOC_OS12G39700-H4) carried the superior haplotype of two candidate genes. The remaining accessions were carriers of superior haplotypes from a single gene (**Figure 4**, **Table 3**).

**Table 3:** *Haplo-Pheno* analysis of candidate gene carrying superior haplotype and functional annotation of the candidate genes.

Sl.no.	Trait	Gene	Gene	Description	Chr	Superior haplotype	Average blast score^*^	Average performance of individuals with other haplotypes
1	Blast	*LOC_Os01g18910*		peroxidase precursor, putative, expressed	1	H29^c^	0.33-1.00	H1^ab^ (0.67-4.67), H10^ab^ (0.33-5.00), H13^a^ (4.33), H16^ab^ (1.33-4.67), H24^ab^ (2.67.4.33), H8^ab^ (0.33-5.00) and H9^b^ (3.67-1.00)
2		*LOC_Os01g24540*			1	H18^b^	1.00-1.33	H151^ab^ (2067-3.67), H254^a^ (4.00-4.23), H26^ab^ (0.33-5), H27^ab^ (0.33-4.00), H273^a^ (4.00-4.33), H557^a^ (2.67-5.00), H6^a^ (3.67-4.33), H81^a^ (3.67-4.00), H98^a^ (4.00-4.33)
3		*LOC_Os01g24640*		transposon protein, putative, CACTA, En/Spm sub-class, expressed	1	H18^b^	1.00-1.33	H151^ab^ (2.67-3.67), H254^a^ (4.00-4.33), H26^ab^ (0.67-5.00), H27^ab^ (0.33-4.00), H273^a^ (4.00-4.33), H557^a^ (3.33-5.00), H6^a^ (3.67-4.33), H81^a^ (3.67-4.67), H98^a^ (4.00-4.33)
4		*LOC_Os01g24750*	*OSPP5*	Ser 2FThr 20protein 20phosphatase 20family 20protein 2C 20putative 2C 20expressed, GO:0004721 - phosphoprotein phosphatase activity	1	H18^b^	1.00-1.33	H151^ab^ (2.67-3.67), H254^a^ (4.00-4.33), H26^ab^ (0.33-5.00), H27^ab^ (0.33-4.00), H273^a^ (4.00-4.33), H557^a^ (2.67-5.00), H6^a^ (3.67-4.33), H81^a^ (3.67-4.67), H98^a^ (4.00-4.30)
5		*LOC_Os04g23960*		retrotransposon protein, putative, Ty3-gypsy subclass, expressed	4	H17^b^	0.67-1.00	H1^a^ (3.00-5.00), H102^a^ (4.00-4.67), H107^a^ (3.00-5.00), H152^a^ (3.33-5.00), H23^a^ (333-4.67), H57^ab^ (1.33-4.00), H59^ab^ (2.67-3.67), H93^a^ (1.00-5.00), H94^a^ (2.67-4.67)
6		*LOC_Os06g21040*		transposon protein, putative, CACTA, En/Spm sub-class	6	H177^b^	1.00-1.00	H1^a^ (3.00 4.67), H166^ab^ (3.33 4.00), H178^a^ (4.00 4.00), H198^ab^ (3.00 3.33), H56^ab^ (0.33 3.67), H57^ab^ (0.33 5.00), H58^ab^ (3.00 4.00), H60^ab^ (1.00 3.33), H8^ab^ (1.00 4.00)
7		*LOC_Os06g30440*	*OsGH3-7*	GO:0009816 - defense response to bacterium, incompatible interaction, GO:0016046 - detection of fungus, GO:0009863 - salicylic acid mediated signaling pathway	6	H33^c^	0.33-0.67	H12^ab^ (1.33 5.00), H126^ab^ (1.33 5.00), H127^ab^ (3.00 3.67), H162^ab^ (2.67 5.00), H163^ab^ (1.00 4.67), H17^ab^ (1.00 5.00), H182^b^ (1.33 3.33), H184^ab^ (1.00 4.67), H89^ab^ (0.33 5.00), H90^ab^ (0.33 5.00), H92^a^ (4.67 4.67), H97^ab^ (3.00 4.67)
8		*LOC_OS12G39700*			12	H3^c^	0.00 to 0.67	H1^ab^ (3.90-5.00), H11^ab^ (4.33-4.33), H112^ab^ (3.60-5.00), H23^ab^ (3.40-5.00), H25^ab^ (3.66-4.33), H26^ab^ (3.70-4.67), H27^ab^ (3.40-5.00), H28^a^ (4.60-4.67), H30^ab^ (3.91-4.67), H31^ab^ (4.00-4.00), H5^bc^ (2.16-3.33), H9^a^ (4.80-5.00)
1	BLB	*LOC_Os02g12660*	*RLCK68*	protein 20kinase 20domain 20containing 20protein 2C 20putative 2C 20expressed	2	H47^b^	1.70 -3.00	H11^a^ (2.12-27.20), H9^ab^ (7.34-11.00), H16^ab^ (2.00-14.62), H17^ab^ (1.72-25.00)
2					2	H39^b^	1.88 -2.06	
3		*LOC_Os05g35490*		hypothetical 20protein	5	H49^c^	1.54-2.34	H111^a^(6.78-27.20), H117(2.00-14.74), H12(5.94-16.90), H16(1.88-14.40), H18(2.26 -7.00), H20(2.60-15.62), H3(3.78-18.60), H31(3.88-18.20), H39(3.72-11.50), H4(1.16-25.00), H49(1.54 -2.34), H64(2.64-12.00), H70(4.46-12.52)
4		*LOC_Os06g05940*		DTA2 2C 20putative 2C 20expressed	6	H112^b^	2.00-3.00	H1^ab^ (2.08-27.20), H11^ab^ (3.28 -4.80), H114^ab^ (2.18-16.40), H17^ab^ (1.52-12.86), H26^ab^ (1.16 -9.60), H3^ab^(1.54-20.60), H48^ab^(1.70-16.60), H6^ab^(2.12-16.90), H7^ab^(1.72 -8.30), H81^a^(1.88-25.00)
5		*LOC_Os06g44340*		retrotransposon 20protein 2C 20putative 2C 20unclassified	6	H29^d^	1.98-3.20	H1^bcd^ (4.46-18.60), H14^bc^ (2.64-18.40), H28^ab^ (5.86-19.20), H36^cd^ (4.30 -4.40), H4^bcd^ (1.16-15.88), H40^a^ (12.00-25.00), H45^bcd^ (2.42-11.96), H5^bcd^ (1.54-20.60), H6^bc^ (3.78-16.40), H9^bcd^ (1.52-23.98)

*Average blast score is recorded 0–5 SES scale (Standard Evaluation System, IRRI, 1996). Scores 0–2 were considered resistant (R), 3 as moderately resistant (MR), and 4-5 as susceptible (S). ^#^Average lesion length <5 cm is resistant, >5-10 cm is moderately resistant, >10-15 cm is moderately susceptible, and >15 cm susceptible (Korinsak et al., 2021); Chr, Chromosome. Different alphabet of small letters denoted in superscript is defined as significantly different groups which authors have got from duncan's test to perform haplo-pheno test.

Eight accessions coming from six diverse countries (Bangladesh, China, India, Myanmar, Pakistan, and Sri Lanka) and spanning four subpopulations (aus, ind1A, ind2, and ind3) were identified as carrying superior haplotypes for BLB resistance. The average lesion length of these accessions ranged from 1.54 to 3.00 cm. Notably, accession MIN ZAO 6::IRGC 63772-1, possessing the superior haplotype of the candidate gene *LOC_Os05g35490*-H49, exhibited the lowest average lesion length of 1.54 cm. Three of the eight accessions—MIN ZAO 6::IRGC 63772-1 (1.54 cm), KUTTA::IRGC 52184-1 (1.70 cm), and KURULU WEE WHITE::IRGC 66518-1 (1.88 cm)—displayed the lowest average lesion lengths for BLB (**Figure 4**, **Table 3**).

The superior haplotypes identified through this study hold promising potential for the development of next-generation disease-resistant varieties via haplotype-based breeding approaches.

## 4 Discussion

Despite the utilization of numerous resistant genes, rice blast, and bacterial blight are continue to be the most devastating and widespread diseases globally. Although more than 100 blast and 42 BLB resistance genes have been identified and employed in resistance breeding programs (Sharma et al., 2012; Alam et al., 2017). Emergence of new pathotypes or races have led to the breakdown of resistance. Consequently, there is a pressing need to discover novel resistance genes or sources to develop durable and sustainable resistant rice lines. The wealth of genetic diversity found in diverse germplasm collections, such as the 3K-RG panel, along with publicly available resequencing data, serves as a valuable resource for identifying novel alleles and donors associated with the trait of interest. GWAS for blast and bacterial blight, using high-density marker information from highly diverse germplasm like the 3K-RG panel, holds tremendous importance in pinpointing the genomic regions associated with these major diseases in rice.

Conventional exploration of novel resistance genes/QTLs has primarily relied on a linkage mapping approach. However, this method has its limitations, including prolonged population development time and the detection of only a few segregating alleles. In contrast, GWAS have emerged as a powerful alternative for mapping genomic regions associated with biotic stress, surpassing biparental or linkage mapping approaches. GWAS involves identifying genomic regions related to biotic stress in a diverse set of germplasm. Over the past few decades, substantial efforts have been dedicated to identifying genes or QTLs that confer resistance to blast and BLB in rice, resulting in numerous noteworthy findings. The genotyping data from the 3K RG project has proven to be a valuable resource for several researchers in pinpointing genomic regions associated with both abiotic and biotic stress in rice (Shi et al., 2017; Phan et al., 2023; Zhang et al., 2017; Zhang et al., 2019). By leveraging the information obtained through GWAS and the comprehensive genotyping data available in the 3K-RG, have made significant strides in understanding and enhancing resistance to biotic stresses, paving the way for the development of more resilient and disease-resistant varieties.

In this study, we focused on a sub-set of rice accessions from the 3K-RG to investigate blast and bacterial blight diseases. Our aim was to identify significant MTAs and unravel the haplotype diversity of candidate genes associated with these diseases. Through an in-house multi-locus GWAS analysis, we discovered a total of 23 MTAs, with 8 linked to blast resistance and 14 to bacterial blight resistance.

What makes our findings particularly intriguing is that all the identified MTAs were novel, with no previously reported genes for blast and bacterial blight within these loci, except for one blast resistance gene, *Pi40*, which was located near MTA-17556914 on chromosome 6. However, when we expanded the search to include sequences within −/+ 100 kb from the significant MTAs, we found four overlapping resistant genes on chromosome 11 associated with bacterial blight: *Xa23* (*LOC_Os11g37620*), *Xa39* (*LOC_Os11g37759*), *Xa10* (*LOC_Os11g37570*), and *Xa46* (*LOC_Os11g37540*). Furthermore, our comprehensive analysis led to the detection of 107 candidate genes on chromosomes 1, 4, 6, 7, 8, 11, and 12 associated with blast resistance and 324 candidate genes on chromosomes 1, 3, 4, 5, 7, 8, 10, 11, and 12 associated with bacterial blight resistance. These findings contribute to a deeper understanding of the genetic basis of blast and bacterial blight resistance, offering valuable insights for the development of improved disease-resistant varieties through targeted breeding programs.

In pursuit of developing durable resistant cultivars, we employed the identification and deployment of superior haplotypes for pest and disease resistance, which is a promising approach. Through haplotype analysis of candidate genes, we successfully identified several superior haplotypes for the target traits, with 8 superior haplotypes for blast resistance and 4 for bacterial blight resistance. Interestingly, four candidate genes (*LOC_Os06g21040, LOC_Os04g23960, LOC_Os12g39700*, and *LOC_Os01g24640*) encoding transposon and retrotransposon proteins (CACTA, En/Spm sub-class, putative, *Ty3-gypsy* subclass) were among those with superior haplotypes. These genes are known to play a crucial role in plant defense responses (Todorovska, 2007). Furthermore, one of the candidate genes, *LOC_Os01g24640*, encodes the peroxidase gene, which has essential functions in the oxidation of various components and is involved in the biosynthesis and degradation of lignin in cell walls. The peroxidase gene is critical in the host plant’s resistance during the basal (PTI) defense response (Yoshida et al., 2003). These findings shed light on the genetic mechanisms underlying the resistance traits and provide valuable insights for developing disease-resistant cultivars by strategically deploying superior haplotypes.

We discovered a superior haplotype of the candidate gene *LOC_Os06g30440* on chromosome 6, which encodes for *OsGH3-7*. This gene plays a crucial role in the salicylic acid (SA)-mediated signaling pathway. SA is a key plant hormone responsible for triggering host responses against microbial pathogens. Specifically, it induces a defense response against biotrophic pathogens like blast pathogens, which rely on living host tissues for their growth and reproduction. On the other hand, the jasmonic acid (JA)-activated defense response is targeted toward wounding and necrotrophic pathogens. Our identification of *LOC_Os06g30440*’s involvement in the salicylic acid-mediated signaling pathway highlights its significance in combating the blast pathogen, which is a biotrophic pathogen. Additionally, on chromosome 1, we identified the gene *LOC_Os01g24750*, which codes for *OSPP5*, a serine-threonine family protein. This protein has been previously reported to play a role in plant defense responses. These findings contribute to a better understanding of the genetic mechanisms involved in the plant’s defense against pathogens and offer potential targets for enhancing disease resistance through genetic manipulation or breeding programs.

Superior haplotypes were identified in four candidate genes associated with bacterial blight (BLB). Upon functional annotation of these genes obtained from RAP-DB, one candidate gene, *LOC_Os02g12660* on chromosome 2, was found to encode a receptor-like cytoplasmic kinase (*RLCK*). *RLCKs* belong to a significant subfamily of proteins that play a crucial role in regulating plant immunity against bacterial and fungal pathogens in various plant species, including rice, tomato, and *Arabidopsis* (AbuQamar et al., 2008; Vij et al., 2008; Ao et al., 2014). In the case of bacterial blight resistance in rice, the function of Receptor-like cytoplasmic kinase has already been reported. RLCKs are involved in PAMP-triggered immunity, a key defense mechanism in plants. Specifically, in rice, the *RLCK* gene *OsRLCK185* encodes an *RLCK* that is directly phosphorylated by the lysine motif-containing PAMP-receptor *OsCERK1*. Suppression of *OsRLCK185* expression has been observed to result in reduced MAP kinase activation and reduced expression of chitin-induced genes *PBZ1* and *PAL1*, which are important components of the plant’s immune response (Yamaguchi et al., 2013; Wang et al., 2017).

In this study, it was found that a *Xanthomonas oryzae* effector called *Xoo1488* can suppress the interaction between *OsRLCK185* and *OsCERK1*, indicating the essential role of the *OsRLCK185/OsCERK1* complex in plant immune responses, particularly PTI (PAMP-triggered immunity). However, the other three candidate genes identified in the study were not directly linked to disease resistance.

Overall, these findings underscore the significance of the identified candidate genes in the context of blast and BLB resistance and offer potential targets for enhancing the plant’s ability to combat these devastating diseases. The study revealed several novel genomic regions (MTAs) associated with blast and BLB diseases, along with their corresponding superior haplotypes. These superior haplotypes could be effectively integrated into a single genetic background using a haplotype-based breeding approach, ultimately leading to the development of varieties with a broader and more effective resistance spectrum against both blast and bacterial blight diseases. This approach holds promise for enhancing rice cultivars’ durability and resilience to these devastating diseases.

## 5 Conclusions

In conclusion, rice blast and bacterial blight poses a global threat to rice yield, making the identification of resistance genes and haplotypes a crucial strategy for disease management. Our GWAS on a diverse set of 147 rice accessions led to the discovery of 23 significant marker-trait associations (9 for blast and 14 for BLB), corresponding to 107 and 210 candidate genes for blast and BLB, respectively. *Haplo-pheno* analysis revealed eight superior haplotypes for blast and five for BLB, with remarkable SES and lesion length scores. Notably, the candidate genes possessing the superior haplotypes are known to play vital roles in plant defense responses. The identified superior haplotypes, sourced from diverse subpopulations and countries, hold promise to be incorporated into a single genetic background through haplotype-based breeding, providing a broader resistance spectrum against blast and bacterial blight diseases.

## Data availability statement

The raw data supporting the conclusions of this article will be made available by the authors, without undue reservation.

## Author contributions

SA: Conceptualization, Project administration, Data curation, Investigation, Methodology, Validation, Writing – original draft. KS: Data curation, Formal analysis, Software, Writing – review & editing. US: Data curation, Formal analysis, Writing – review & editing, Methodology. MS: Writing – review & editing, Resources. GL: Resources, Writing – review & editing. PS: Formal analysis, Resources, Writing – review & editing, Conceptualization, Data curation, Methodology, Project administration. VS: Conceptualization, Project administration, Writing – review & editing, Funding acquisition, Supervision.
