# Supplementary material for: Superior haplotypes towards the development of blast and bacterial blight-resistant rice
Source: Front Plant Sci. 2024 Feb 28;15:1272326. doi: 10.3389/fpls.2024.1272326 (PMC10932988; doi:10.3389/fpls.2024.1272326)
Supplement: Supplementary file 2 [file DataSheet_1.docx]

***Supplementary Material***

**Superior haplotypes towards development of blast and bacterial blight resistant lines in rice**

Shamshad Alam^1^, Krishna Tesman Sundaram^1^, Uma Maheshwar Singh^2^, Madamshetty Srinivas Prasad^3^, Gouri Sankar Laha^3^, Pallavi Sinha^1,*^, Vikas Kumar Singh^1,*^

^1^Rice breeding Innovation, International Rice Research Institute (IRRI) South Asia Hub, ICRISAT campus, Hyderabad, India.

^2^Rice breeding Innovation, International Rice Research Institute (IRRI) South Asia Regional Centre, Varanasi, Uttar Pradesh

^3^Department of Plant Pathology, Indian Council of Agriculture Research-Indian Institute of Rice Research (ICAR-IIRR), Hyderabad, India

**Keywords:** Rice, Blast, BLB, GWAS, *M. oryzae*, *X. oryzae*

***Author for Correspondence**: [v.k.singh@irri.org](mailto:v.k.singh@irri.org); [p.sinha@irri.org](mailto:p.sinha@irri.org)

**Supplementary Figures**

**
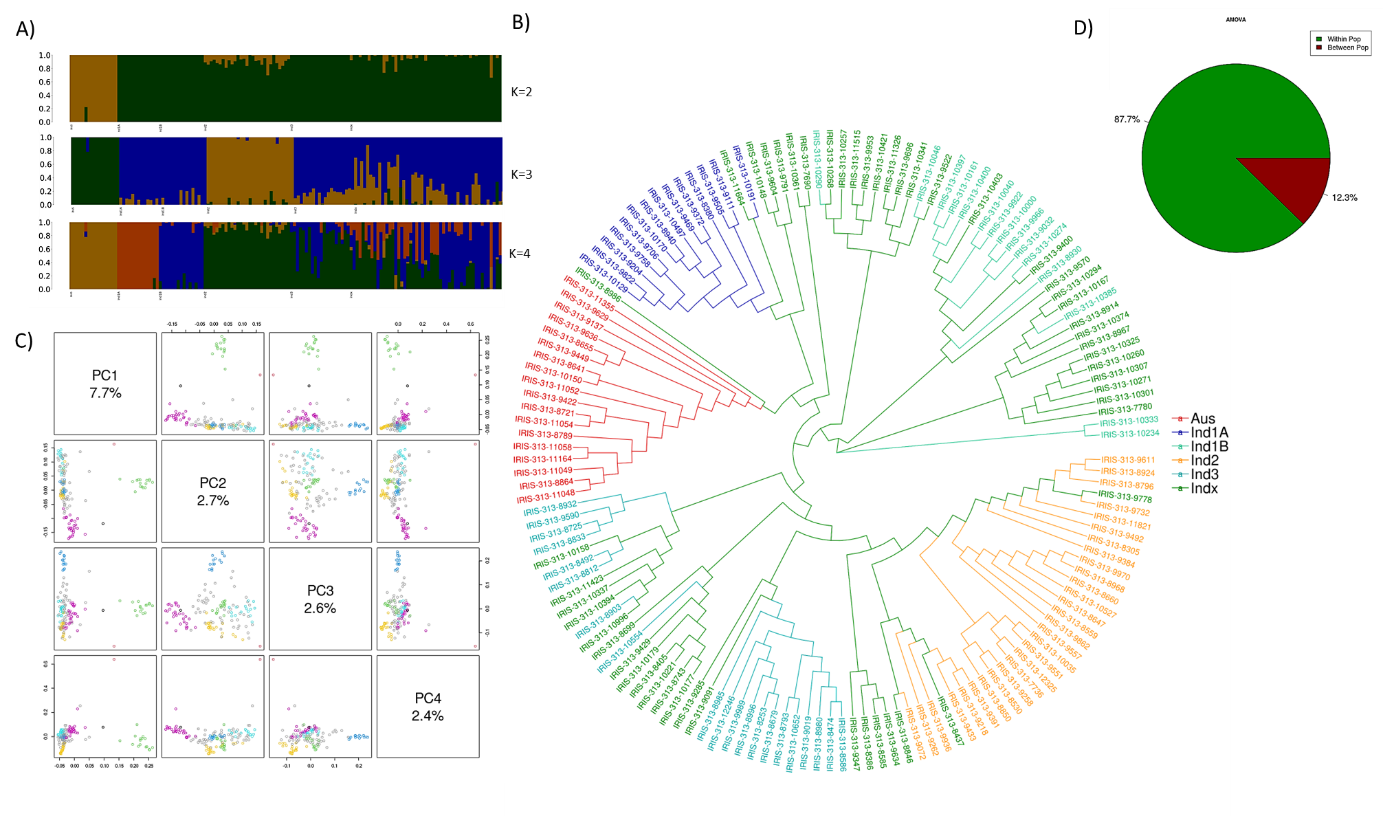
**

**Supplementary Figure 1.** The population diversity of 147 blast selected population showing a divergence within and between the population. (A) Admixture analysis from K value 2 to 3 representing six different sub groups (i) aus, (ii) ind1A, (iii) ind1B, (B) Cladogram tree showing the relationships between the subgroups analysed from the neighbour joining methods. (C) The Principal Components explain 15% of total variance in the entire population while each component variance is explained. (D) Analysis Molecular variance gives the explanation of variance between and within the groups and samples. 87% variance is observed within the samples and 12% between groups explains the selection between the genetic material over the years.

**
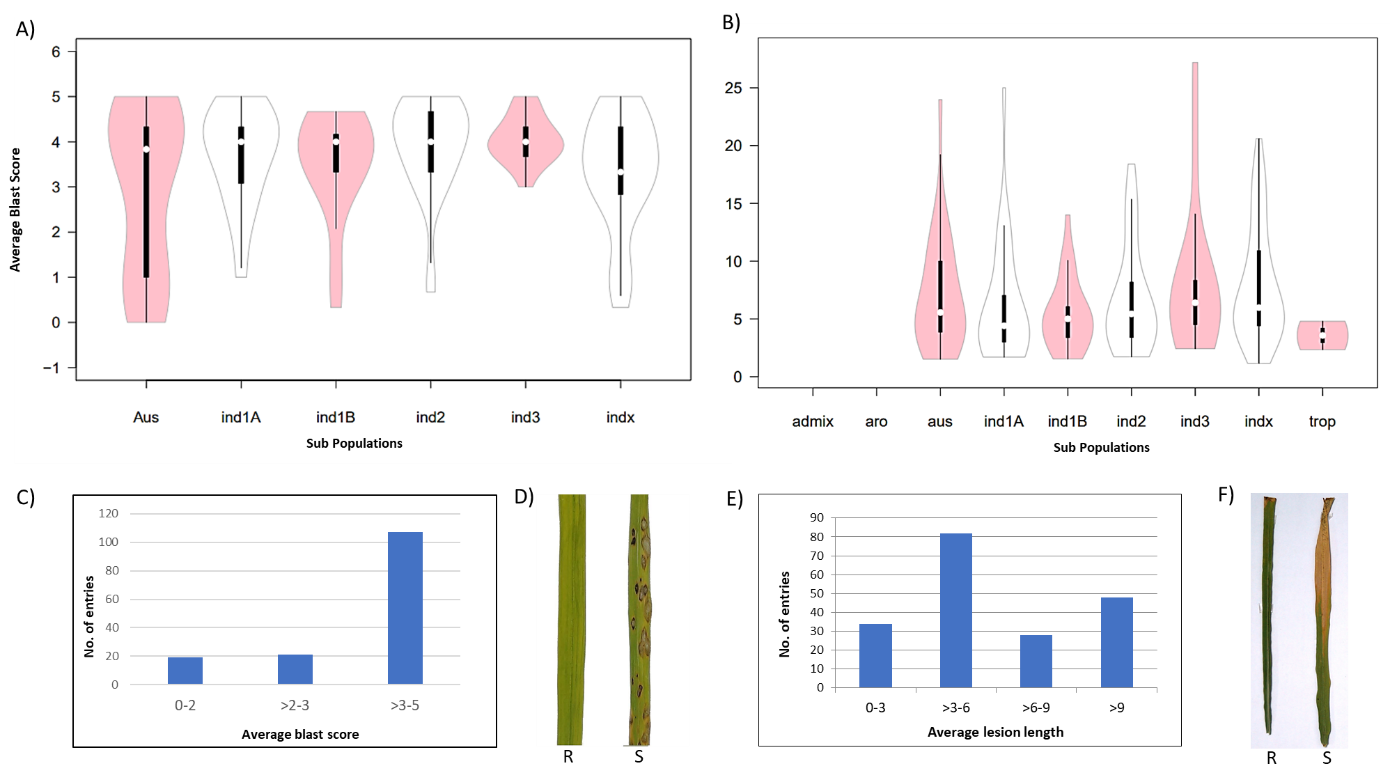
**

**Supplementary Figure 2.** Phenotyping distribution of blast and BLB resistance in 147 accessions of 3K RG panel. A) & B) The violin plots show the phenotypic distribution of the different subpopulation of 3K RG panel for the targeted traits. C) & E) Frequency distribution of blast and BLB showing phenotypic variation in the subset of 3K rice genome panel. D) & F) Reaction of susceptible and resistant parent for blast and BLB

**
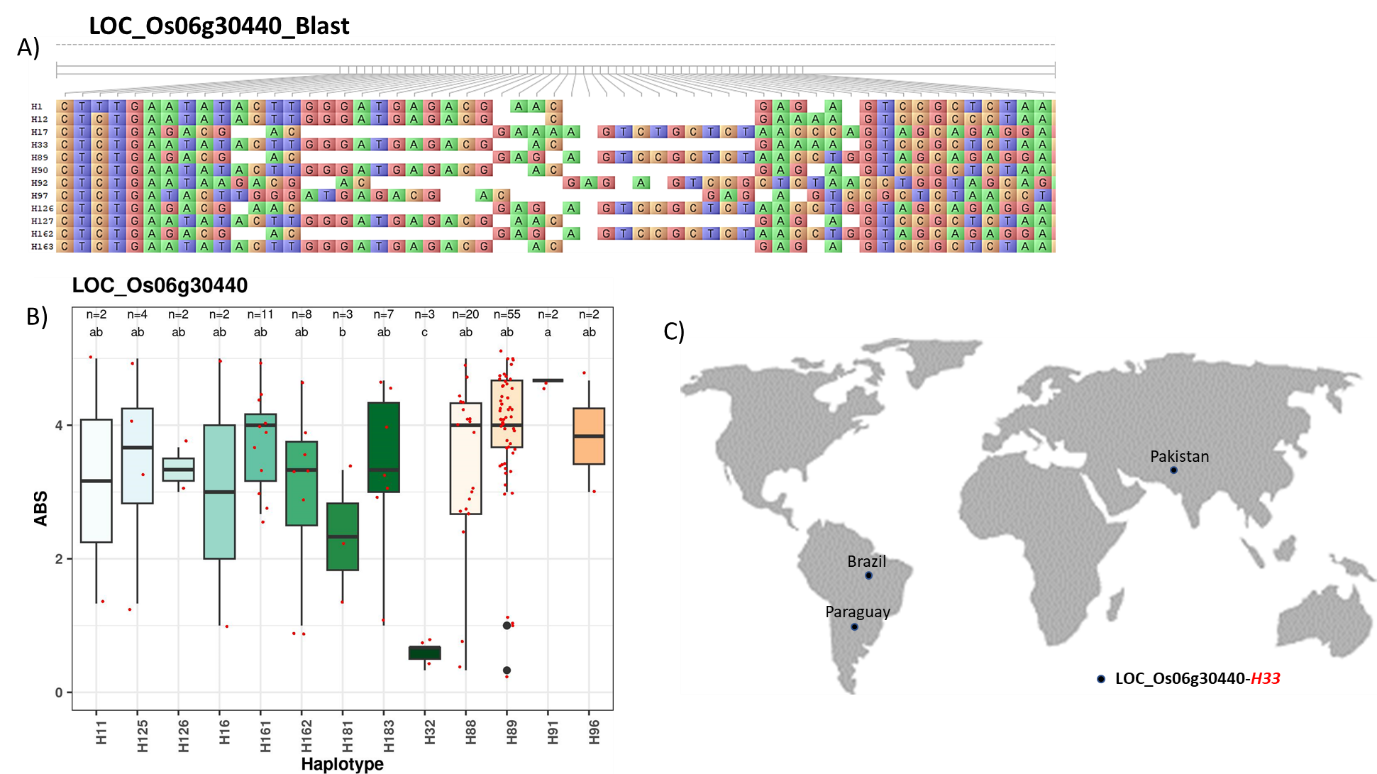
**

**Supplementary Figure 3.** Haplotype analysis of LOC_Os06g30440 across the subset panel. A) Haplotypic variation of LOC_Os06g3044, a gene associated with blast resistance. B) Boxplot showing variation in blast resistance among 147 accessions of 3K RGP C) The geographical distribution of superior haplotype

**
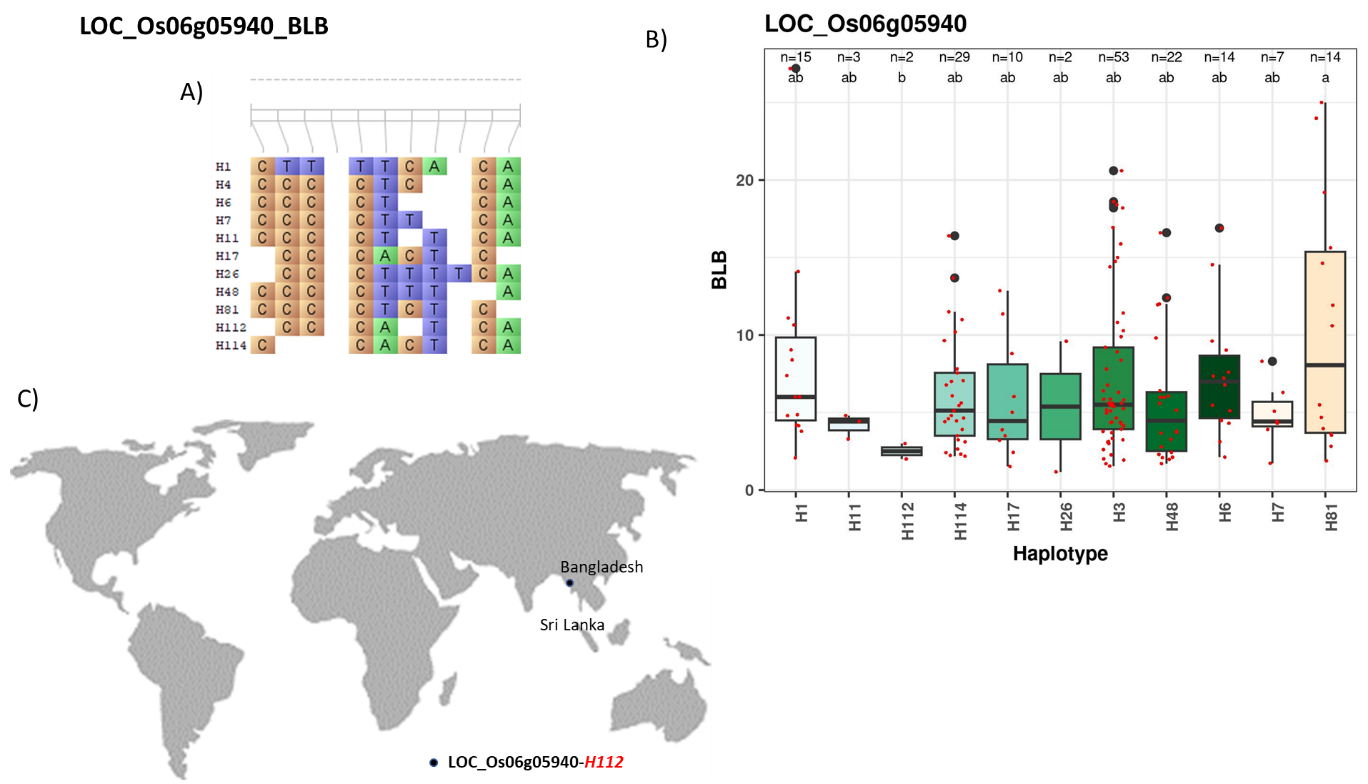
**

**Supplementary Figure 4.** Haplotype analysis of LOC_Os06g05940 across the subset panel. A) Haplotypic variation of LOC_Os06g05940, a gene associated with BLB resistance. B) Boxplot showing variation in BLB resistance among 147 accessions of 3K RGP C) The geographical distribution of superior haplotype


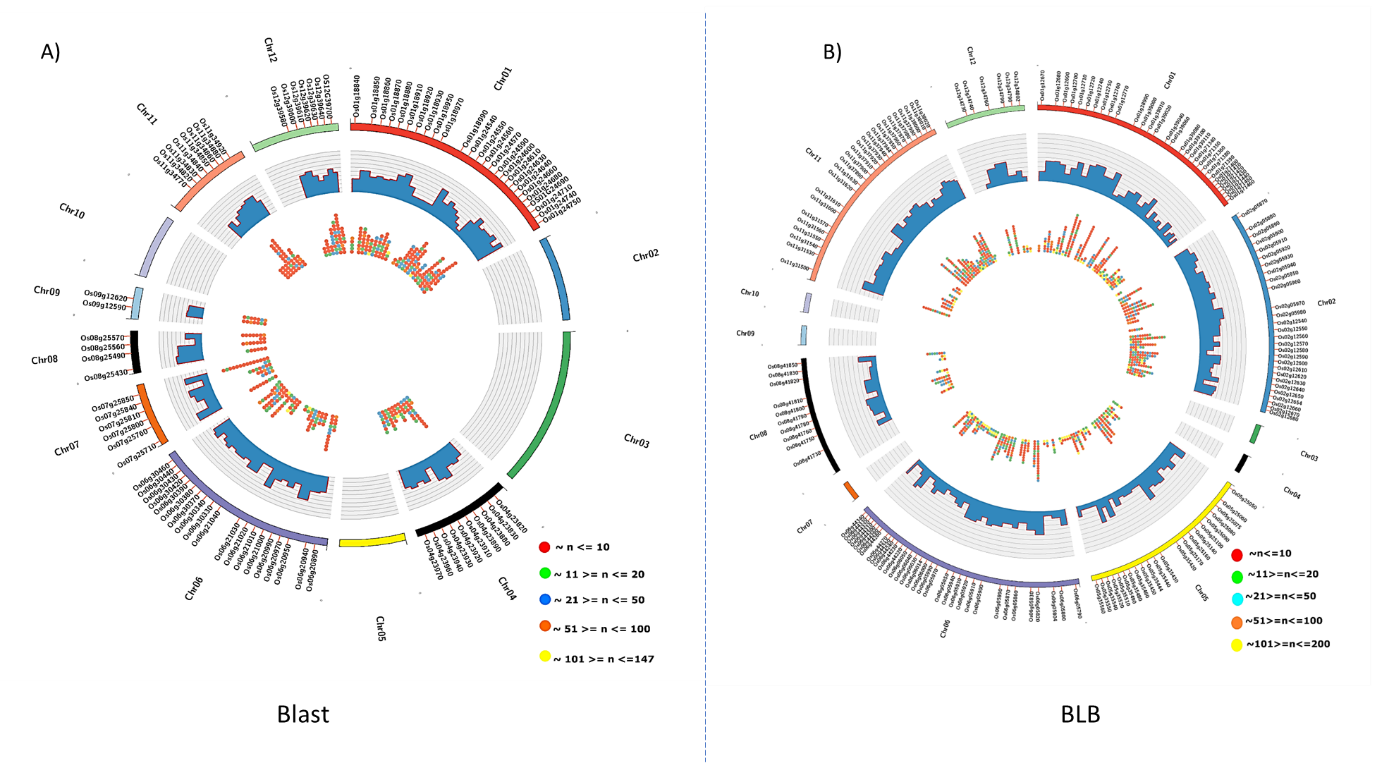


**Supplementary Figure 5.** Circos plot showing the haplotype diversity of 107 candidate genes associated with blast resistance (A) and 210 candidate genes associated with BLB resistance (B). The outer shell represents the candidate genes present on different chromosomes. The second outer most shell represent the haplotype diversity of each candidate genes. The innermost shell represents the number of haplotype of each candidate genes. n represent the number of germplasm in that particular haplotype
